# What is the cause of the opthalmoplegia in this young child? What treatment is necessary?

**DOI:** 10.1002/ccr3.38

**Published:** 2014-02-12

**Authors:** Karim Kassam, Bhavin Visavadia

**Affiliations:** Northwick Park HospitalLondon, HA1 3UJ, United Kingdom

**Keywords:** Blowout, children, fracture, white-eye.

## Abstract

**Key Clinical Message:**

Not all orbital fractures are associated with clinical signs of swelling, ecchymosis, and subconjunctival hemorrhage. The “white-eyed” blowout fracture is more commonly seen in children and is associated with entrapment of the extraocular muscles. Early surgical intervention is indicated and it must have been in the differential diagnosis of the head injury patient with opthalmoplegia.

## Case History

This young child is unable to look up in the right eye due to a mechanical entrapment of the inferior rectus muscle. He has a “white-eye” blowout fracture. Classic signs of this type of fracture are: diplopia, limitation on a gaze of which the direction is dependent upon the trapped extraocular muscle(s). The “white-eye” phenomenon refers to the lack of clinical evidence of soft tissue trauma, such as swelling, ecchymosis, or subconjunctival hemorrhage. Bradycardia, hypotension, headache, nausea, and vomiting are all culminations of the oculocardiac reflex that is more pronounced in children. Early surgical intervention is required in order to prevent necrosis of any entrapped muscles and subsequent visual impairment. This child uneventually had the muscle released with no complications thereafter. Clinicians should be aware of this condition and must not confuse it with a head injury.

**Figure 1:**
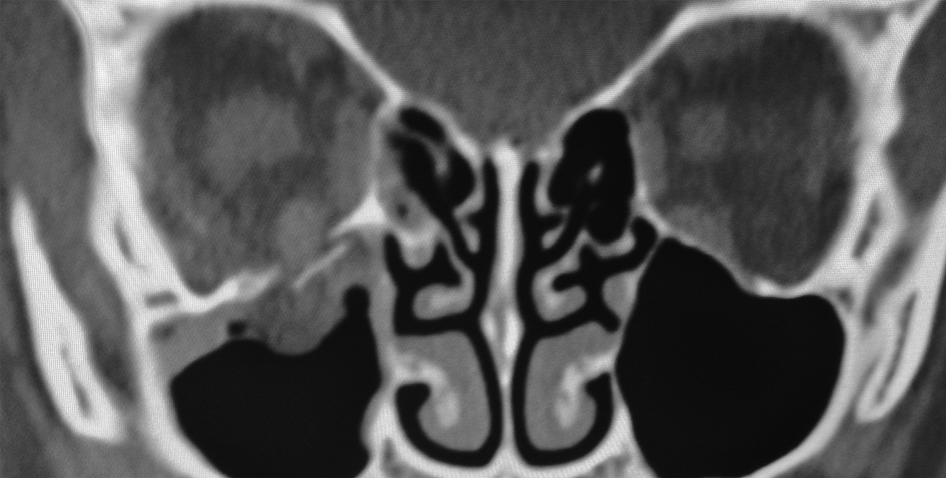
CT showing fracture of the orbital floor with muscle herniating into the defect.

**Figure 2:**
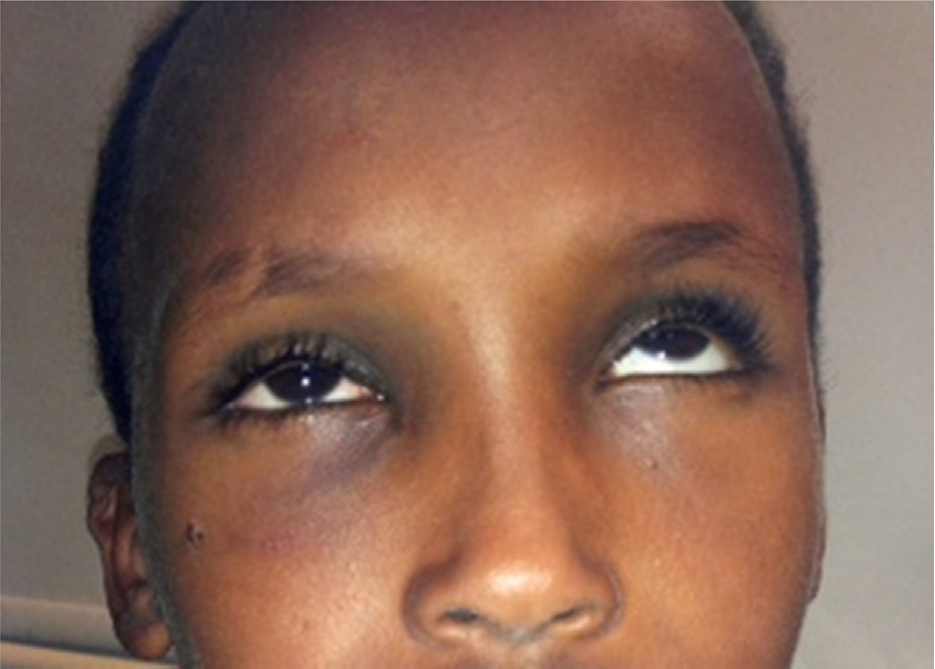
This child is unable to look up in the right eye due to a mechanical entrapment of the inferior rectus muscle.

**Figure 3:**
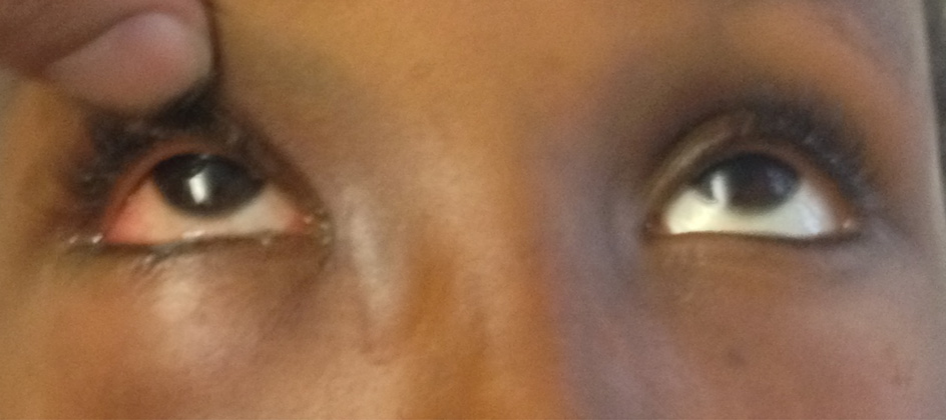
Unrestricted movement of the right eye following release of muscle.

## Supporting Information

**Video S1.** Pre-operative assessment of “white eye” blowout.

**Video S2.** Post-operative assessment.

